# Correction: Bromodomain inhibition of the transcriptional coactivators CBP/EP300 as a therapeutic strategy to target the IRF4 network in multiple myeloma

**DOI:** 10.7554/eLife.19432

**Published:** 2016-07-14

**Authors:** Andrew R Conery, Richard C Centore, Adrianne Neiss, Patricia J Keller, Shivangi Joshi, Kerry L Spillane, Peter Sandy, Charlie Hatton, Eneida Pardo, Laura Zawadzke, Archana Bommi-Reddy, Karen E Gascoigne, Barbara M Bryant, Jennifer A Mertz, Robert J Sims

Conery AR, Centore RC, Neiss A, Keller PJ, Joshi S, Spillane KL, Sandy P, Hatton C, Pardo E, Zawadzke L, Bommi-Reddy A, Gascoigne KE, Bryant BM, Mertz JA, Sims RJ. 2016. Bromodomain inhibition of the transcriptional coactivators CBP/EP300 as a therapeutic strategy to target the IRF4 network in multiple myeloma. *eLife* **5**:e10483. doi: 10.7554/eLife.10483.

Published January 05, 2016

Owing to a production error the published Supplementary files 1 and 2 were incorrect. Supplementary file 1 was published with the legend for Supplementary file 2 and a file from another eLife article was published with the legend for Supplementary file 1.

The correct Supplementary files 1 and 2 are available here.

Supplementary file 1. Includes qPCR primer sequences and UPL probe numbers for RT-qPCR experiments described in the manuscript.

Supplementary file 2. Indicates the source of all cell lines used, as well as results of mycoplasma testing throughout the course of these studies.

The article has now been corrected accordingly.

## Additional files

Supplementary file 1. Includes qPCR primer sequences and UPL probe numbers for RT-qPCR experiments described in the manuscript.

Supplementary file 2. Indicates the source of all cell lines used, as well as results of mycoplasma testing throughout the course of these studies.

